# Tailored liposomal nanotraps for the treatment of Streptococcal infections

**DOI:** 10.1186/s12951-021-00775-x

**Published:** 2021-02-15

**Authors:** Hervé Besançon, Viktoriia Babiychuk, Yu Larpin, René Köffel, Dominik Schittny, Lara Brockhus, Lucy J. Hathaway, Parham Sendi, Annette Draeger, Eduard Babiychuk

**Affiliations:** 1 Institute of Anatomy, University of Bern, 3012 Bern, Switzerland; 2 Institute for Infectious Diseases, University of Bern, 3001 Bern, Switzerland

**Keywords:** Infection, Streptococcus, Toxin, Liposome, Nanotrap

## Abstract

**Background:**

Streptococcal infections are associated with life-threatening pneumonia and sepsis. The rise in antibiotic resistance calls for novel approaches to treat bacterial diseases. Anti-virulence strategies promote a natural way of pathogen clearance by eliminating the advantage provided to bacteria by their virulence factors. In contrast to antibiotics, anti-virulence agents are less likely to exert selective evolutionary pressure, which is a prerequisite for the development of drug resistance. As part of their virulence mechanism, many bacterial pathogens secrete cytolytic exotoxins (hemolysins) that destroy the host cell by destabilizing their plasma membrane. Liposomal nanotraps, mimicking plasmalemmal structures of host cells that are specifically targeted by bacterial toxins are being developed in order to neutralize-by competitive sequestration-numerous exotoxins.

**Results:**

In this study, the liposomal nanotrap technology is further developed to simultaneously neutralize the whole palette of cytolysins produced by *Streptococcus pneumoniae*, *Streptococcus pyogenes* and *Streptococcus dysgalactiae subspecies equisimilis*-pathogens that can cause life-threatening streptococcal toxic shock syndrome. We show that the mixture of liposomes containing high amounts of cholesterol and liposomes composed exclusively of choline-containing phospholipids is fully protective against the combined action of exotoxins secreted by these pathogens.

**Conclusions:**

Unravelling the universal mechanisms that define targeting of host cells by streptococcal cytolysins paves the way for a broad-spectrum anti-toxin therapy that can be applied without a diagnostic delay for the treatment of bacterial infections including those caused by antibiotic-resistant pathogens.

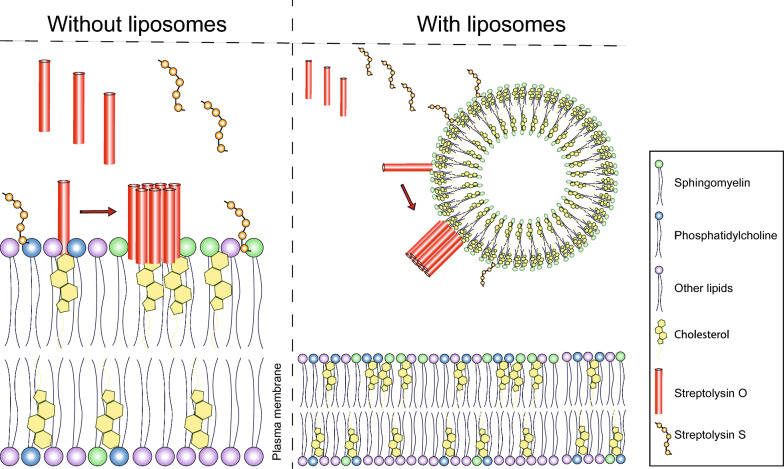

## Introduction

Infectious diseases are responsible for a staggering 15 million deaths annually, accounting for more than half of the deaths in low income countries [1, 2]. In developed countries, infectious diseases re-emerge as a major health threat; the aging population is becoming increasingly immunocompromised by chronic disease, chemotherapy, or organ transplantation and these patients inevitably enter healthcare environments, where antibiotic resistant pathogens are prevalent [2, 3]. Furthermore, evolution-driven bacterial resilience and almost exhausted options for the development of new antibiotic classes, in combination with longer-living population, force the pharmacological industry to abandon antibiotics in favor of more profitable medications against chronic diseases.

In an effort to identify new, non-antibiotic approaches for the treatment of bacterial infections, bacterial virulence factors have come into focus as pharmacological targets. Virulence factors are specific agents produced by bacterial pathogens that allow them to survive within the hostile environment of the targeted organism [2, 4, 5]. An anti-virulence treatment eliminates the advantage provided by targeted virulence factors, thus promoting bacterial clearance by the immune system. Since anti-virulence agents do not interfere with bacterial survival, they do not apply selective evolutionary pressure on the pathogens and are unlikely to foster the development of resistance [2, 4, 5]. Moreover, anti-virulence strategies are complementary to antibiotics and offer a chance to improve treatment for a better outcome.

Secreted exotoxins are a class of virulence factors produced by both Gram-positive and Gram-negative bacteria [6–11]. Bacterial exotoxins have a multifactorial role in the evolution of infections, causing tissue and organ damage, facilitating bacterial dissemination, disabling the host’s immune defense, and prompting highly damaging immune (over)responses. Exotoxins either initiate toxic signaling cascades within the cytoplasm of a host cell or act as cytolysins by perforating the plasma membrane and thereby disrupting the protective barrier of the cell. In any case, they must initially interact with a component of the cell membrane. Many cytolysins bind specifically to the cholesterol- and sphingolipid-rich regions of the plasma membrane known as lipid rafts [7, 8, 12, 13].

*Streptococcus pneumoniae* is the leading cause of bacterial pneumonia; *Streptococcus pyogenes* (Group A Streptococcus, GAS) and *Streptococcus dysgalactiae subspecies equisimilis* (Group G Streptococcus, GGS) cause diseases ranging from uncomplicated pharyngitis to severe, life-threatening invasive illnesses such as necrotizing fasciitis, pneumonia and sepsis [1, 14].

Streptococcal exotoxins pneumolysin (PLY) and streptolysin O (SLO) belong to the large family of structurally and functionally related cholesterol-dependent cytolysins (CDCs); other prevalent pathogens producing CDCs include *Clostridium* spp., *Listeria* spp. and *Bacillus* spp. During the progress of infection, CDCs are released by the bacteria as soluble monomers that bind to the plasmalemmal cholesterol of host cells, assemble in oligomeric pores and perforate the plasmalemmal lipid bilayer [12, 13]. Analyses of the mechanisms responsible for the CDC-induced virulence revealed that high toxin doses are rapidly cytocidal, but low doses are tolerated because a limited number of plasma membrane lesions can be resealed [15–18]. However, even in the resealed cells, an initial membrane perforation induces a homeostatic dysbalance, which provokes the pathological activation of a broad variety of intracellular signaling pathways.

Taking advantage of cholesterol binding, we and others have developed liposomal nanotraps, composed of purified lipids, or nanosponges, containing a polymeric core wrapped in a cell-derived lipid bilayer, to neutralize bacterial exotoxins [15, 19–21]. Liposomal nanotraps are empty vesicular structures made up of one or more lipid bilayers. They provide an environment, which mimics the in vivo toxin target, in order to divert the toxins from attacking a host cell. Nanotraps saturated with cholesterol neutralized multiple cholesterol-binding toxins, including not only several CDCs, but also cytolysins belonging to different toxin families with different modes-of-action such as α-hemolysin from *Staphylococcus aureus* and phospholipase C from *Clostridium perfringens* [15]. In vivo, liposomal nanotraps rescued infected mice from deadly bacteremia and pneumonia induced by *S. pneumoniae* and *S. aureus* [15]. We have also shown that the whole toxin secretomes of *S. pneumoniae* and *S. aureus* contain additional cytotoxic activities that are different from hemolytic activities of PLY and α-hemolysin and that require nanotraps composed exclusively of sphingomyelin for their neutralization. Recently, the sphingomyelin-binding toxin of *S. aureus* was identified as phenol-soluble modulin α3 [21].

Streptococcal cytolysins are released in particularly high amounts after bacterial lysis caused by antibiotic therapy [22, 23]. Therefore, these toxins can cause widespread damage and lead to fatal complications even after successful antibiotic treatment. We have shown that low doses of liposomal nanotraps augmented the effects of antibiotics in a *S. pneumoniae* mouse model [15]. Recently, the safety and efficacy of CAL02 (Combioxin S.A., Switzerland)—a pharmacological agent that is based on our liposomal formulations [15] was assessed in a first-in-human study as an add-on therapy to antibiotics in patients with severe community-acquired pneumonia caused by *S. pneumoniae* [24]. CAL02, which efficiently neutralizes pneumococcal toxins, proved to be safe and well tolerated; moreover, the CAL02 group displayed a clear trend to a faster resolution of the infection compared to placebo [24].

*Streptococcus pneumoniae* generates PLY, whereas GAS and GGS produce SLO as unique, specie-specific CDCs [12, 13]. Yet, in addition to SLO and in contrast to *S. pneumoniae*, GAS and GGS secrete another cytolysin, streptolysin S (SLS), a small peptide that belongs to the class of thiazole/oxazole-modified microcins (TOMMs) [25]. Other prevalent pathogens producing SLS-like toxins are *Clostridium* spp., *Listeria* spp. and *Staphylococcus* spp. [25].

Our study provides mechanistic insights into the targeting of host cells by streptococcal cytolysins and describes novel targets for PLY and SLO. Unravelling the mechanisms that govern SLS binding to the plasmalemma of host cells enabled us to expand our liposomal nanotrap technology in order to simultaneously neutralize all secreted streptococcal cytolysins irrespective of their species or strain specificity.

## Results

**Fig. 1:**
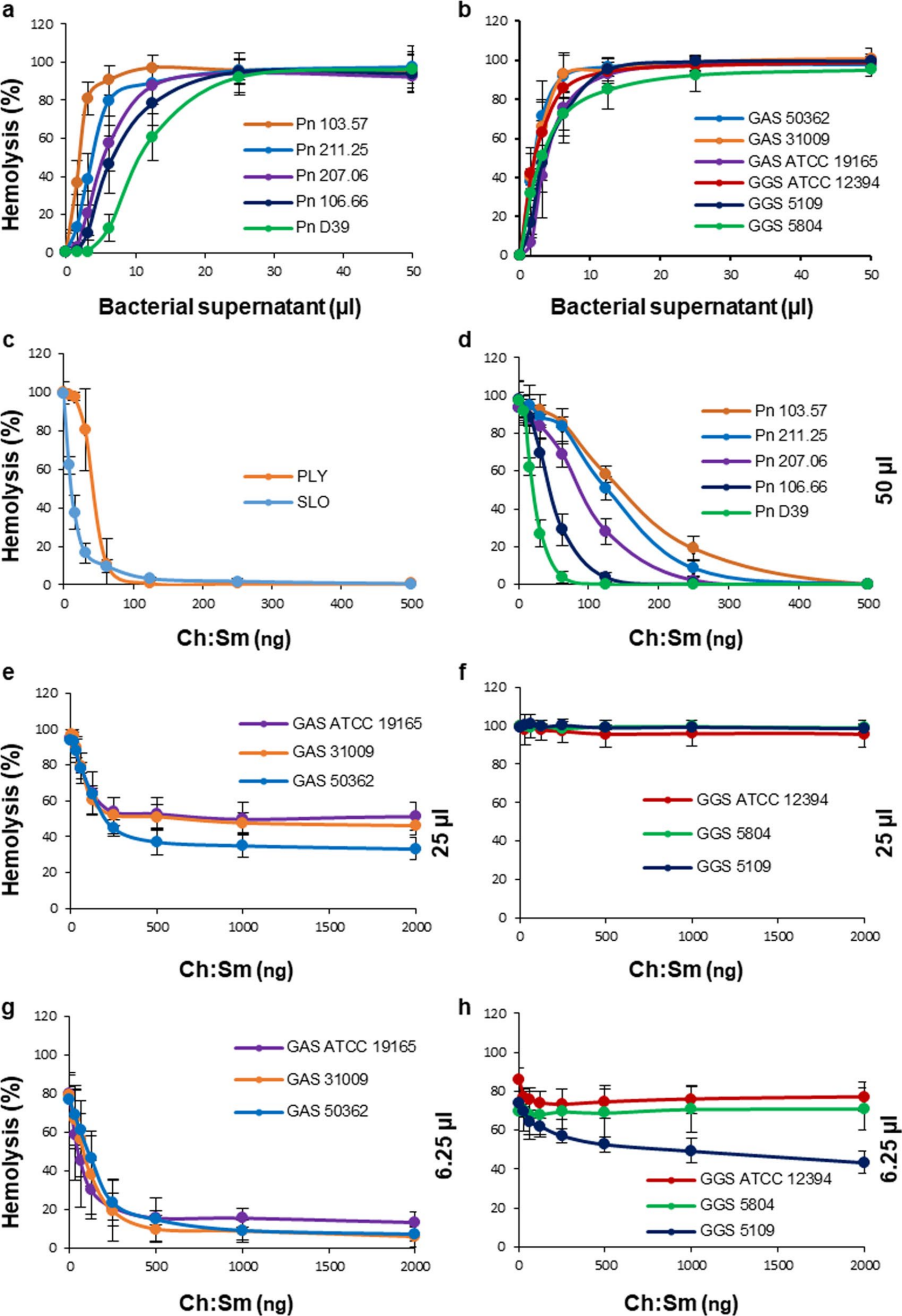
Neutralization of hemolysins secreted by *S. pneumoniae*, *S. pyogenes* and *S. dysgalactiae subspecies equisimilis* by liposomes saturated with cholesterol. Filtered supernatants obtained from cultures of *S. pneumoniae* (Pn), *S. pyogenes* (GAS) and *S. dysgalactiae subspecies equisimilis* (GGS) display potent hemolytic activities (**a**, **b**). Ch:Sm-liposomes (Ch = 66 mol/%) completely neutralize hemolytic activities of recombinant PLY and SLO as well as the activities of Pn supernatants (**c**, **d**). These liposomes provide only partial protection against GAS and GGS supernatants (**e–h**). Error bars = Mean ± SD. N ≥ 3

All strains of *S. pneumoniae*, GAS and GGS used in this study possessed potent hemolytic activities that were comparable between species (Fig. 1a, b). Dependent on the strain, the amounts of bacterial culture supernatants required for the complete hemolysis (100% hemolytic activity) ranged between 6.25 µl and 25 µl (Fig. 1a, b). To evaluate the contribution of individual hemolytic activities (toxins) to the total hemolytic activity of individual strains, all bacterial supernatants were used both at non-saturating (≤ 100% hemolytic activity) and at saturating amounts (≥ 200% hemolytic activity).

Liposomes (50–100 ng) saturated with cholesterol (Ch) and containing sphingomyelin (Sm) as bilayer-forming lipid, Ch:Sm-liposomes; Ch = 66 mol/%; 100 ng ≈ 885 nM total lipid or 584 nM Ch) completely inhibited the hemolytic activities of recombinant PLY and SLO (Fig. 1c), which are unique CDCs produced by pneumococci and streptococci, respectively [12, 13, 15].

Likewise, Ch:Sm-liposomes (50–500 ng) completely neutralized the hemolytic activities of all *S. pneumoniae* supernatants used at their saturating amounts (50 µl = 200–800% hemolytic activity, Fig. 1d).

In contrast, Ch:Sm-liposomes provided only partial protection against saturating amounts of GAS supernatants and did not provide any protection against saturating amounts of GGS supernatants (25 µl = 200–400% hemolytic activity, Fig. 1e, f).

The partial protection against GAS supernatants was characterized by an initial protective effect occurring at low amounts of the Ch:Sm-liposomes (up to 250–500 ng) without further protection at higher amounts (Fig. 1e). This biphasic protection, characterized by a clear neutralization plateau, is reminiscent of the presence of two distinct hemolysins in GAS supernatants, of which only one is inhibited by the Ch:Sm liposomes. Indeed, GAS and GGS produce two hemolysins: a cholesterol-dependent cytolysin (SLO) and a thiazole/oxazole-modified microcin (SLS) [25]. Our results suggest that the cholesterol (Ch:Sm)-neutralizable hemolytic activity of GAS/GGS strains is mediated by the cholesterol-dependent cytolysin, SLO, whereas the cholesterol-insensitive activity is, most likely, mediated by SLS.

When bacterial supernatants were used at their non-saturating amounts (6.25 µl = 70–90% hemolytic activity, Fig. 1g), Ch:Sm-liposomes provided almost complete protection against the hemolytic activities of all GAS supernatants (Fig. 1g). This suggests that the Ch:Sm-neutralizable SLO is the major hemolysin of GAS. It was further evident that all GAS supernatants possessed Ch:Sm-insensitive hemolytic activity (presumably SLS); albeit its contribution to the total hemolytic activity was low (~ 10% of the total 70–90% hemolytic activity) at these experimental conditions (Fig. 1g).

Compared to GAS, the Ch:Sm-insensitive hemolytic activity was much more evident in all GGS supernatants (Fig. 1f, h). At the saturating amounts (25 µl = 200–400% hemolytic activity) this hemolytic activity of GGS supernatants was sufficient to enforce complete hemolysis even at the conditions when the SLO-mediated hemolysis was inhibited by the Ch:Sm-liposomes (Fig. 1f). At the non-saturating amounts (6.25 µl = 70–90% hemolytic activity), minor Ch:Sm-sensitive hemolytic activities of SLO, present in GGS 5109 and GGS ATCC 12394 supernatants, manifested themselves in a slight Ch:Sm-dependent drop in the hemolysis at the beginning of concentration curves, followed by a neutralization plateau (Fig. 1h). For the GGS 5804 strain, no Ch:Sm-dependent hemolytic activity of SLO was detected even at these experimental conditions (Fig. 1h).

**Fig. 2:**
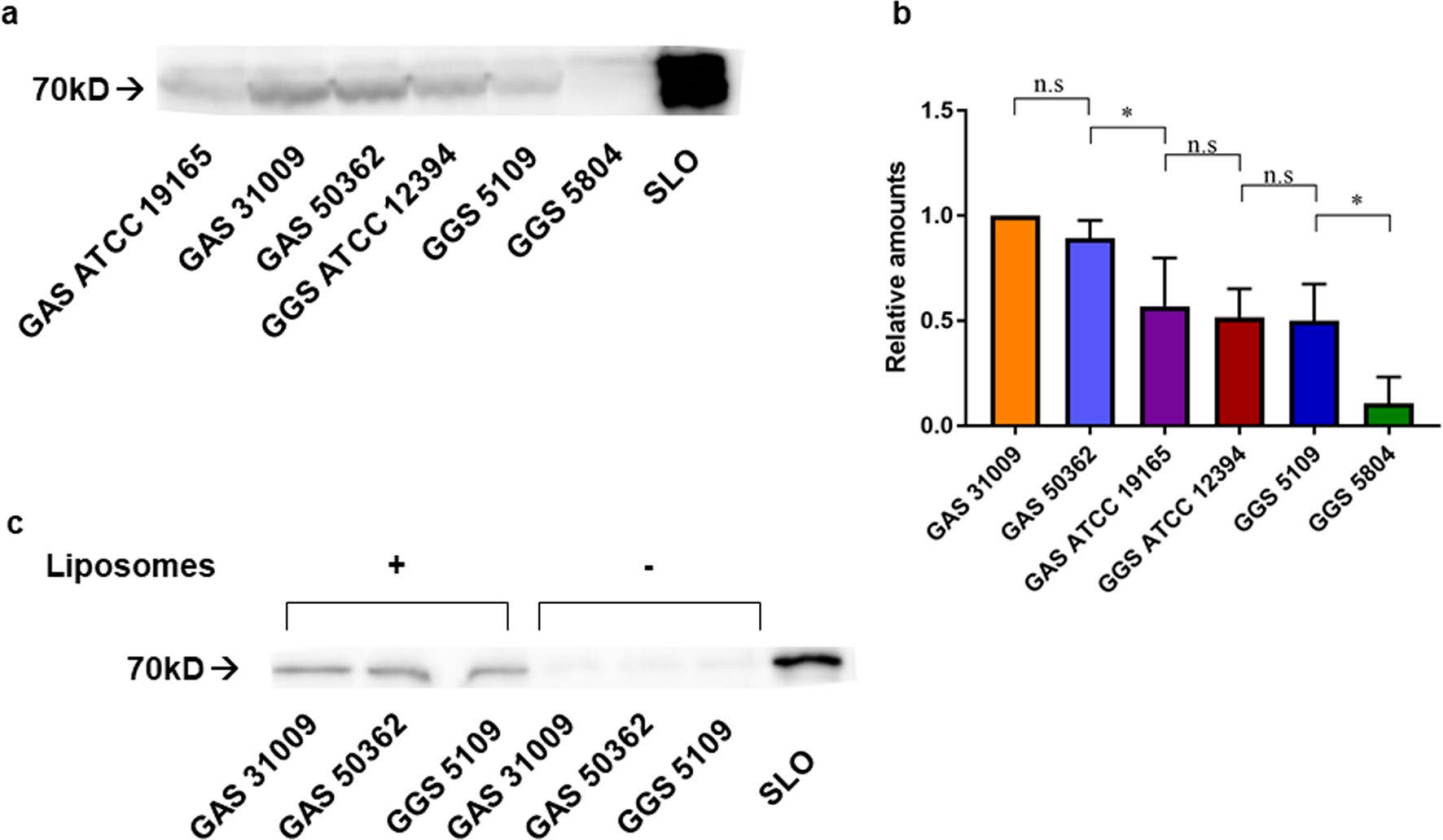
Detection of secreted SLO in filtered bacterial culture supernatants of GAS/GGS strains by Western blotting analysis (**a**). Relative amounts of SLO in GAS/GGS supernatants are shown normalized to the GAS 3109 strain. Error bars = Mean ± SD. N = 3 (**b**). Liposomal pull-down assay reveals direct binding of SLO secreted by GAS 31009, GAS 50362 and GGS 5109 to Ch:Sm-liposomes (**c**)

Western blotting analysis confirmed the presence of SLO in GAS and GGS supernatants (Fig. 2a). Generally, compared to GGS, GAS strains secreted more SLO (Fig. 2b), which is in line with our activity data (Fig. 1e, h).

Ch:Sm liposomes neutralize CDCs by direct binding. [7, 8, 12, 13, 15] Direct binding of SLO present in streptococcal supernatants to Ch:Sm-liposomes was confirmed by a liposomal pull-down assay, followed by Western blotting analysis of the resulting pellets (Fig. 2c).

SLS is a small, non-immunogenic peptide, which prevents its identification by Western blotting. Therefore, to confirm that SLS was responsible for the Ch:Sm-insensitive hemolytic activity in GAS/GGS supernatants, we used its specific inhibitor, Trypan Blue [25].

**Fig. 3:**
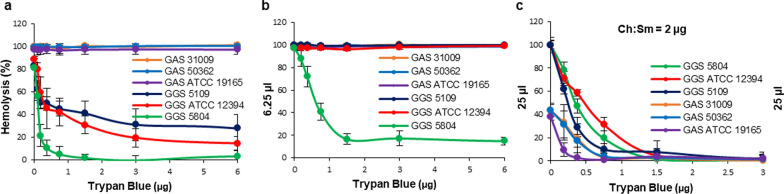
Inhibition of hemolysins secreted by GAS and GGS supernatants by Trypan Blue. Trypan Blue, a selective inhibitor of SLS, provides partial protection against hemolytic activities of GGS supernatants and no protection against GAS supernatants (**a**, **b**). However, the full neutralization of GAS/GGS hemolysins is achieved by the combination of Trypan Blue with Ch:Sm-liposomes (**c**). Please note that in **c** Ch:Sm-liposomes (invariable amount; 2 µg) are present in all experimental conditions (varying amounts of Trypan Blue). As a result, GAS supernatants display already diminished hemolytic activity (~ 50% hemolysis) even in the absence of Trypan Blue, whereas GGS strains are fully hemolytic at these conditions. Error bars = Mean ± SD. N ≥ 3

Trypan Blue inhibited the Ch:Sm-insensitive hemolytic activities of all GGS strains, when their supernatants were used at the non-saturating amounts (Figs. 1h and 3a). At these experimental conditions, Trypan Blue showed no protection against the hemolytic activity of GAS strains that preferentially rely on Ch:Sm-sensitive SLO for their hemolysis (Figs. 1g and 3a). At the saturating amounts of bacterial supernatants, Trypan Blue inhibited the hemolytic activity of the GGS 5804 strain only, since in all other strains the remaining, Trypan Blue-insensitive, SLO-mediated hemolytic activity was sufficient to cause complete hemolysis (Fig. 3b). This experiment also revealed that while the GGS 5804 strain relied mostly on the Trypan Blue-sensitive SLS for its hemolytic activity, it still displayed a weak hemolytic activity of SLO, which was not inhibited by Trypan Blue (~ 10% of the total 400% hemolytic activity).

When used in combination, Ch:Sm-liposomes (inhibition of SLO) and Trypan Blue (inhibition of SLS) completely neutralized the hemolytic activities of all GAS and GGS strains even at the saturating amounts of their supernatants (Fig. 3c).

Taken together, our results imply that *S. pneumomiae* relies on PLY for its hemolytic activity, whereas GAS as well as GGS secrete both SLO and SLS. Cholesterol-sensitive SLO is the major hemolysin of GAS, whereas GGS rely to a greater extent on the cholesterol-insensitive SLS for their hemolytic activity.

The combined action of Ch:Sm-liposomes and Trypan Blue completely neutralized the hemolytic activities of all GAS and GGS strains (Fig. 3c). However, the therapeutic potential of Trypan Blue is uncertain. The great advantage of nanotraps for the neutralization of bacterial toxins is that all components of liposomal nanotraps are naturally occurring lipids that are present at high concentrations in the host organism and hence are neither toxic nor immunogenic. Therefore, we next assessed whether—analogous to the neutralization of SLO—the liposomal lipid composition could be adapted to neutralize SLS.

Interactions between SLS and various phospholipids were reported earlier [26]. For any membrane-damaging toxin, it is a prerequisite to enter into a direct interaction with lipids of the plasmalemmal lipid bilayer at the final stage of its toxic action. Individual lipid species are not randomly distributed across the bilayer. [29] Whereas the inner leaflet contains preferentially phosphatidylethanolamine, phosphatidylserine and phosphatidylinositol, the outer leaflet (the surface) of the plasmalemmal lipid bilayer is enriched in choline-containing phospholipids: sphingomyelin and phosphatidylcholine (PC). Therefore, next we tested whether liposomes composed of either Sm or PC were capable of neutralizing SLS.

**Fig. 4:**
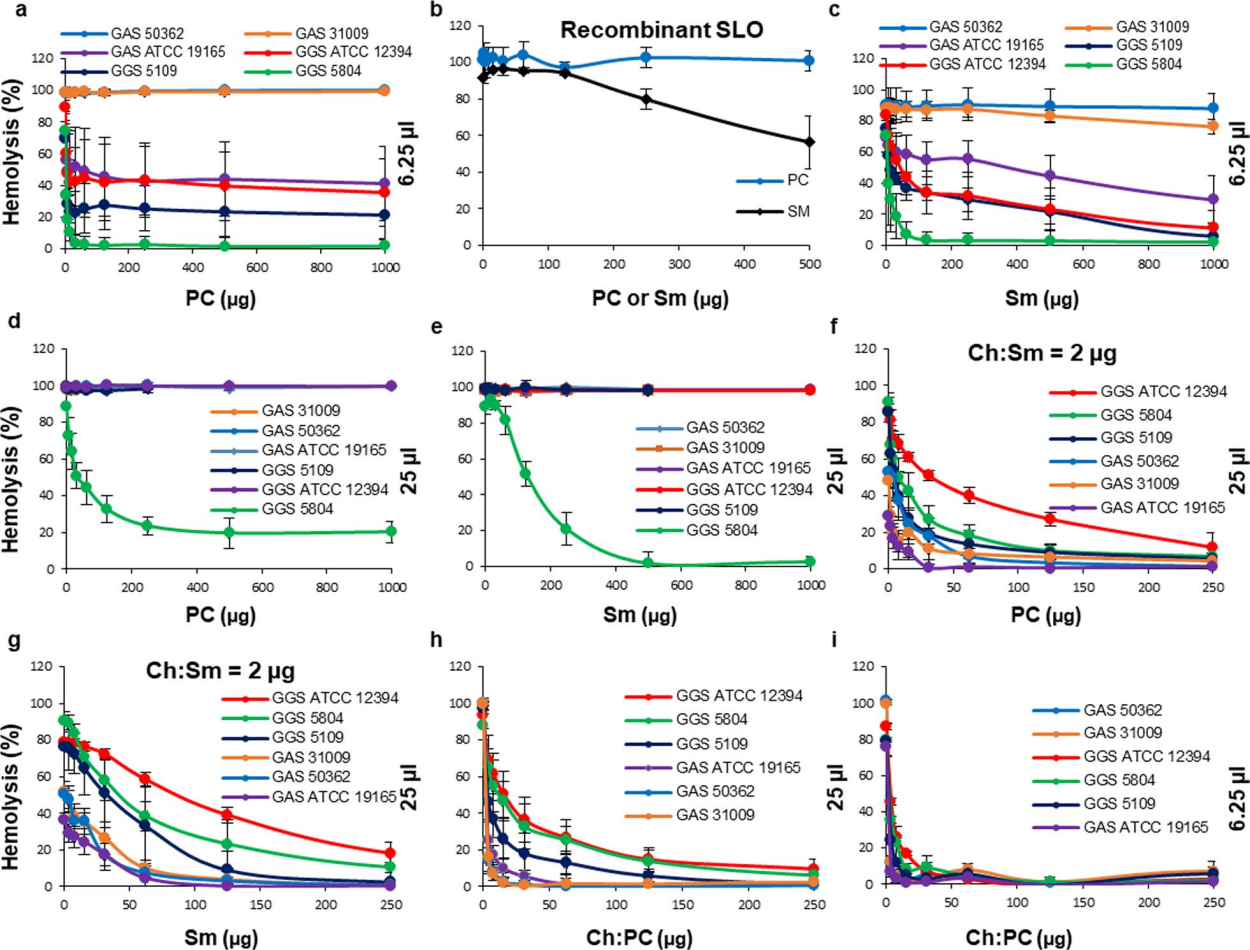
Inhibition of secreted hemolysins of GAS and GGS supernatants or recombinant SLO by liposomes composed of PC or Sm. Liposomes composed of PC provide partial protection against hemolytic activities of all GGS and GAS ATCC supernatants and no protection against GAS 50362 and GAS 31009 supernatants (**a**, **d**). Sm-liposomes inhibit recombinant SLO, whereas PC-liposomes provide no protection (**b**). Liposomes composed of Sm provide full protection against GGS 5804 supernatant, partial protection against GGS 5109, GGS ATCC 12394 and GAS ATCC 19165 supernatants and no protection against GAS 50362 and GAS 31009 supernatants (**c**, **e**). However, the full neutralization of GAS/GGS hemolysins is achieved by the combination of either PC- or Sm-liposomes with Ch:Sm-liposomes (**f**, **g**). Likewise, combined within single liposome, Ch and PC (Ch:PC-liposomes) provide full protection against hemolytic activities of all GAS/GGS supernatants (**h**, **i**). Error bars = Mean ± SD. N ≥ 3

Figure 4a shows that, when the supernatants were used at their non-saturating amounts (70–100% hemolytic activity), PC-liposomes (~ 10 µg) completely inhibited the hemolytic activity of mostly SLS-producing GGS 5804 supernatant and strongly reduced the activities of two other GGS supernatants as well as that of GAS ATCC 19165 supernatants. As expected, cholesterol-free PC-liposomes inhibited neither the hemolytic activities of mostly SLO-producing GAS 50362 and GAS 31009 supernatants (Fig. 4a) nor that of recombinant SLO (Fig. 4b). Therefore, our data suggest that PC-liposomes are capable of inhibiting SLS, whereas they do not affect SLO, whose activity manifests itself as a non-inhabitable plateau of hemolysis observed when the amounts of PC-liposomes exceed 10 µg (Fig. 4a).

Similar to PC-liposomes, Sm-liposomes featured initial, rapid phase of inhibition affecting GGS 5804, GGS 5109, GGS ATCC 12394 and GAS ATCC 19165 supernatants but not GAS 50362 or GAS 31009 supernatants (Fig. 4c). However, in contrast to PC-liposomes, their inhibitory effect was not saturated and almost complete inhibition of all GGS supernatants was achieved, albeit at high amounts of the liposomes (Fig. 4c). Likewise, at their high amounts, Sm-liposomes strongly inhibited recombinant SLO (~ 100% hemolytic activity; (Fig. 4b). It should be noted, however, that the efficacy of SLO inhibition by the Sm-liposomes was approximately 1000 times lower than that of Ch:Sm-liposomes (Figs. 1c and 4b).

At the high saturating amounts of bacterial supernatants (400–800% hemolytic activity), PC- and Sm-liposomes neutralized only the hemolytic activity of the GGS 5804 strain (Fig. 4d, e) since the remaining SLO activity was sufficient to cause complete hemolysis by all other streptococcal supernatants. The residual SLO activity of the GGS 5084 strain manifested itself by an incomplete protection by PC-liposomes but not by Sm-liposomes, due their SLO-neutralizing ability (Fig. 4d, e).

Most important, the combination of cholesterol-containing liposomes (for SLO neutralization) with liposomes composed of choline-containing lipids (for the neutralization of SLS) fully inhibited the hemolytic activities of all streptococcal strains even at the high saturating amounts of bacterial supernatants (Fig. 4f, g). Also, when combined within the same liposome, Ch in combination with either PC (Ch:PC-liposome, Fig. 4h, i) or Sm (Ch:Sm-liposome, not shown) fully neutralized the hemolytic activities of all streptococcal strains.

PC and Sm contain choline as a head group. Originating from natural sources, both lipids contain a variety of acyl chains of different length and saturation status. In addition to sphingosine, egg Sm (Avanti Polar Lipids) was almost exclusively composed of saturated acyl chains with 16:0 being the major species (86%). PC (Avanti Polar Lipids) was more heterogeneous and consisted of a number of different lipid species with acyl chains that varied in length and saturation. We did not observe significant differences in toxin-sequestration between soy PC (major acyl chain specie-polyunsaturated 18:2 (63%); ~ 20% saturated acyl chains) and egg PC (major acyl chain specie-monounsaturated 18:1 (32%); ~ 45% saturated acyl chains) (data not shown). In order to determine the precise nature of the toxin-sequestering targets, we performed experiments with synthetic PCs of defined acyl chain compositions.

**Fig. 5:**
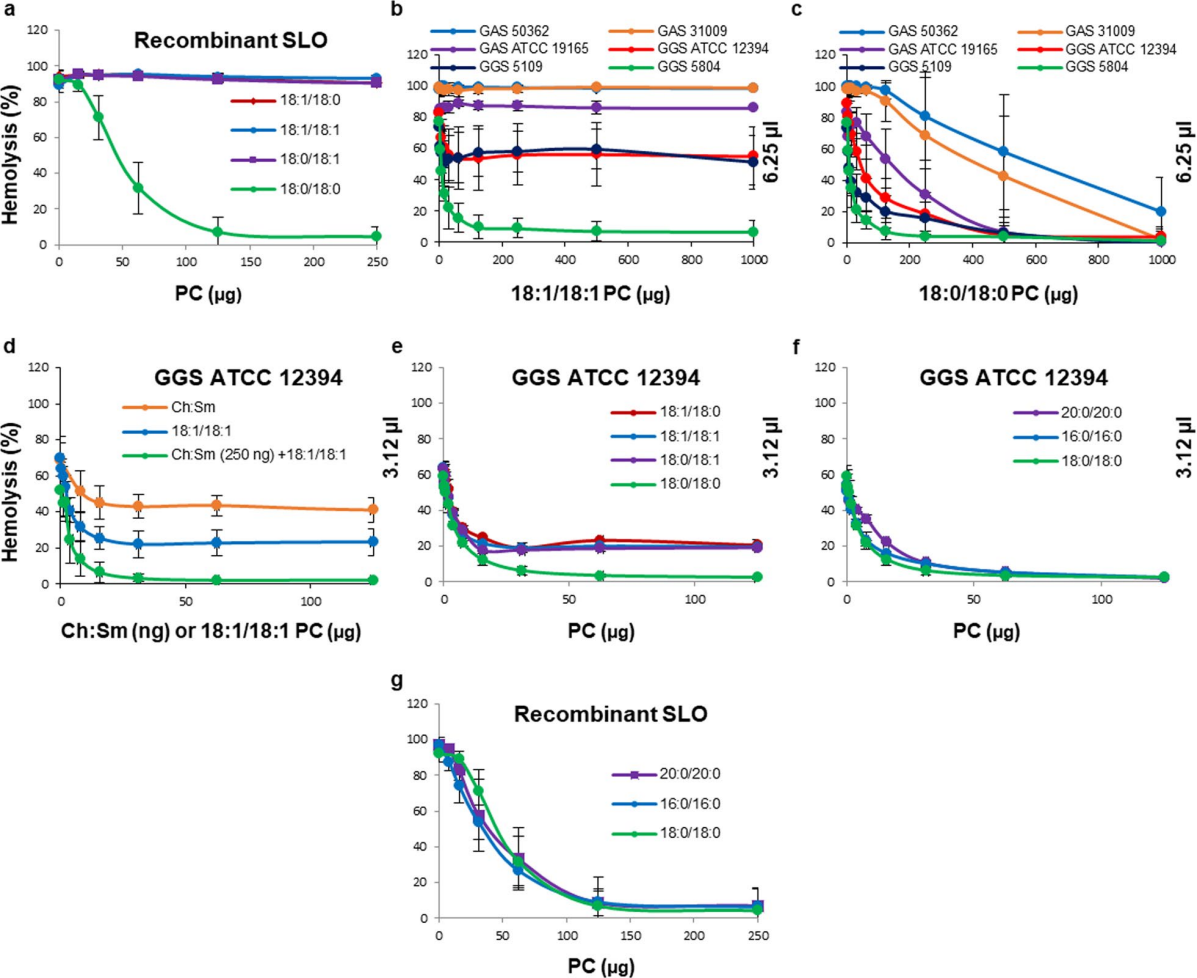
Inhibition of secreted hemolysins of GAS and GGS supernatants or recombinant SLO by liposomes composed of synthetic PCs with saturated or monounsaturated acyl chains. PC-liposomes containing fully saturated acyl chains provide full protection against recombinant SLO; whereas liposomes containing a monounsaturated acyl chain at any position are unable to neutralize recombinant SLO (**a**). Liposomes composed of monounsaturated PC provide full protection against hemolytic activity of GGS 5804 supernatant, partial protection against GGS 5109, GGS ATCC 12394 supernatants and no protection against any GAS supernatants (**b**). Liposomes composed of saturated PC provide full protection against all GAS/GGS supernatants (**c**). Ch:Sm-liposomes or liposomes composed of monounsaturated PC provide only partial protection against hemolytic activity of GGS ATCCC 12394 supernatant; however, their combination is fully protective (**d**). Liposomes containing fully saturated acyl chains provide full protection against hemolytic activity of GGS ATCC 12394 supernatant; whereas liposomes containing a monounsaturated acyl chain at any position are only partially protective (**e**). The length of saturated acyl chains does not affect the liposomal neutralization of the hemolytic activity of GGS ATCC 12394 supernatant (**f**) or recombinant SLO (**g**). Error bars = Mean ± SD. N ≥ 3

Similar to mostly unsaturated PC from natural sources (Fig. 4), liposomes composed from monounsaturated 18:1/18:1 PC did not neutralize the hemolytic activity of recombinant SLO (Fig. 5a), nor were they effective against supernatants obtained from GAS strains that preferentially secrete SLO (Fig. 5b). However, 18:1/18:1 PC-liposomes efficiently neutralized supernatants of GGS strains that preferentially produce SLS. As a result, at the non-saturating amounts of supernatants (6.25 µl), 18:1/18:1 PC-liposomes provided complete protection against the hemolytic activity of the GGS 5804, partial protection against GGS 5109 and GGS ATCC 12394 strains and no protection against any of the GAS strains (Fig. 5b).

Liposomes composed of fully saturated 18:0/18:0 PC were as efficient as 18:1/18:1 PC-liposomes against supernatants of SLS-producing GGS strains (Fig. 5b and Fig. 5c, initial phases of inhibition). However, in contrast to 18:1/18:1 PC-liposomes, liposomes composed of fully saturated 18:0/18:0 PC were active against recombinant SLO (Fig. 5a). Therefore, liposomes composed from saturated 18:0/18:0 PC were able to neutralize all streptococcal supernatants in a biphasic way, with the initial, highly efficient protection phase attributable to the inhibition of SLS, followed by a second, less efficient phase of SLO inhibition (Fig. 5c). Similar pattern of inhibition was observed for mostly saturated liposomes composed from natural Sm (Fig. 4c). However, 18:0/18:0 PC-liposomes were more effective in the neutralization of recombinant SLO (Fig. 5a) than liposomes composed of natural Sm (sphingosine + 16:0 acyl chain) (Fig. 4b). As a consequence, 18:0/18:0 PC-liposomes were more efficient than Sm-liposomes in the neutralization of SLO-containing GGS and, especially, GAS supernatants (Fig. 5c and Fig. 4c).

The relative, individual contribution of SLO and SLS to the total hemolytic activity of GGS ATCC 12394 supernatant at its non-saturating amounts (3.12 µl; total activity ~ 60%) is shown in Fig. 5d. Both 18:1/18:1 PC-liposomes (inhibition of SLS) and Ch:Sm-liposomes (inhibition of SLO) provided only partial protection against the hemolytic GGS ATCC 12394 supernatant. At these experimental conditions the contribution of SLS to the total activity of the supernatant (inhibited by 18:1/18:1 PC-liposomes) was approximately two times higher than that of SLO (inhibited by Ch:Sm-liposomes). The full protection against the hemolytic activity of GGS ATCC 12394 supernatant was achieved by a combination of the two liposomes. Saturation status of acyl chains did not influence the liposomal sequestration of SLS present in GGS ATCC 12394 supernatant (identical initial phase of protection at low liposome concentration, Fig. 5d). In contrast, only liposomes composed exclusively of fully saturated PC were active against SLO present in GGS ATCC 12394 supernatant (full protection, Fig. 5e), whereas liposomes composed of PC containing at least one unsaturated acyl chain either in position sn1 or sn2 were not effective (partial protection, Fig. 5e). Likewise, only liposomes composed exclusively of fully saturated PC were active against recombinant SLO (Fig. 5a). The length of acyl chains did not have any effect, either on sequestration of SLS and SLO present in GGS ATCC 12394 supernatant (Fig. 5f) or recombinant SLO (Fig. 5g).

**Fig. 6:**
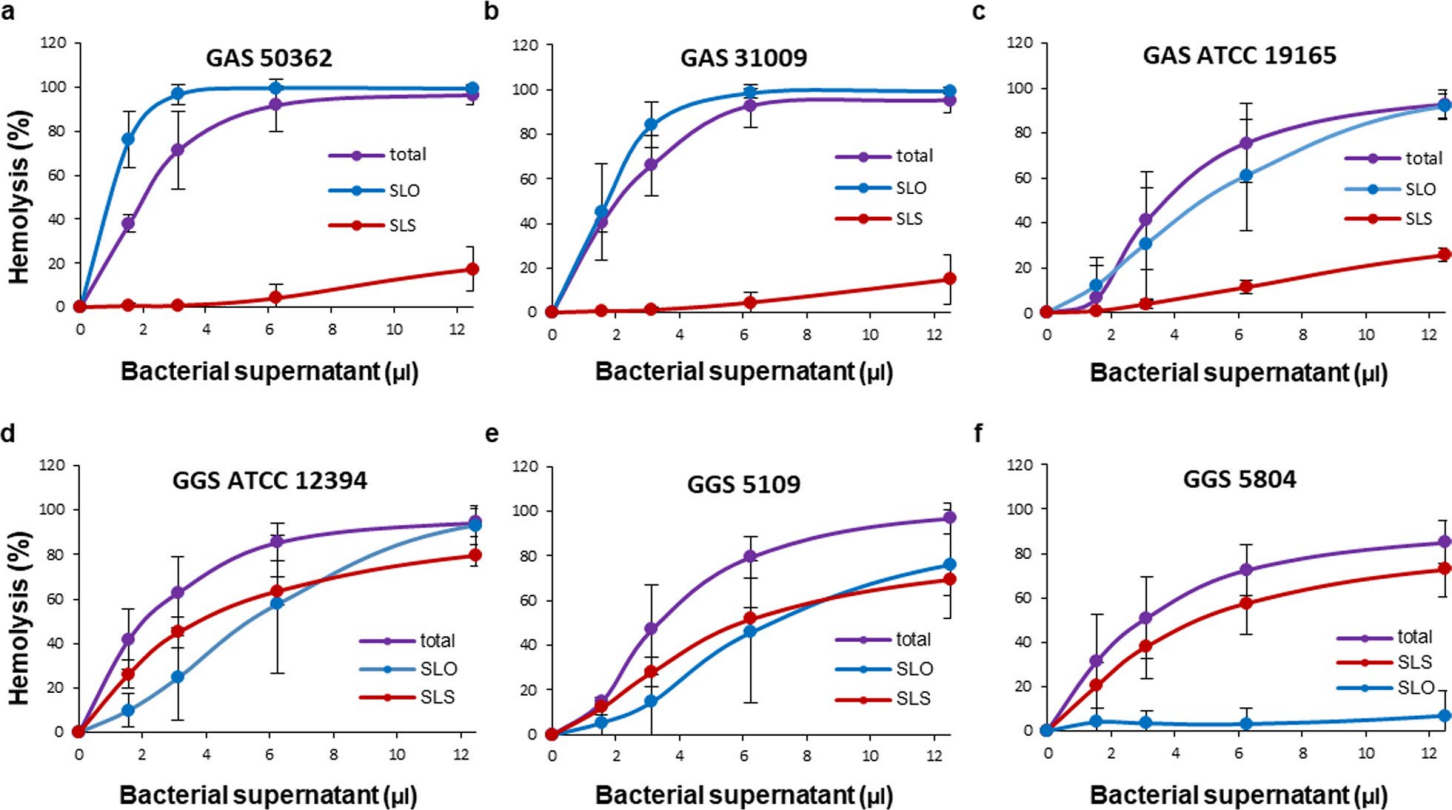
Selective inhibition of SLO and SLS reveals individual dynamics of the toxins and their contribution to the total hemolytic activity of GAS and GGS supernatants (**a–f**). Total: total hemolytic activity of GAS/GGS supernatants. SLO: remaining hemolytic activities of the supernatants after selective inhibition of SLS by 18:1/18:1 PC-liposomes (1000 µg). SLS: remaining hemolytic activities of the supernatants after selective inhibition of SLO by Ch:Sm-liposomes (2 µg). Error bars = Mean ± SD. N ≥ 3

In order to evaluate the individual dynamics of SLO and SLS and their contribution to the total hemolytic activities of GAS and GGS over a range of streptococcal supernatant concentrations, their activities were individually inhibited by either Ch:Sm-liposomes (for inhibition of SLO) or by liposomes composed of 18:1/18:1 PC (inhibition of SLS) (Fig. 6). This experiment revealed that the hemolytic activities of GAS depended almost exclusively on SLO. After inhibition of SLO, the SLS-dependent hemolysis was apparent only at high, saturating amounts of the supernatants, i.e. at conditions at which all erythrocytes would have already been lysed by SLO in the non-treated supernatants (Fig. 6a–c). The opposite was observed for the GGS 5804 strain, which relied almost exclusively on SLS (Fig. 6f). The hemolytic activities of two other GGS strains (GGS ATCC 12394 and GGS 5109) were equally dependent on SLO and SLS (Fig. 6d, e). The dependence of the hemolytic activities of either toxin on their concentrations was non-linear and the toxins appeared to possess different modes of action characterized by different degrees of cooperativity (Fig. 6d, e). For the GGS ATCC 12394 and GGS 5109 strains that rely on both toxins for their hemolytic activity, this difference in the mode-of-action resulted in a complex, concentration-dependent, individual contribution of SLO and SLS towards the total hemolytic activity. At a low concentration of bacterial supernatants, SLS activity prevailed over that of SLO, whereas at higher concentrations SLO became more active than SLS (Fig. 6d, e). It also appears that at least for GAS supernatants, SLO and SLS compete for the binding sites on the plasmalemma of targeted cells. As a result, the total hemolytic activity of GAS 50362 and presumably GAS 31009 and GAS ATCC 19165 (no statistical significance is reached in the last two experiments) is lower than the activity of SLO alone recorded after inhibition of SLS by 18:1/18:1 PC (Fig. 6a–c).

**Fig. 7:**
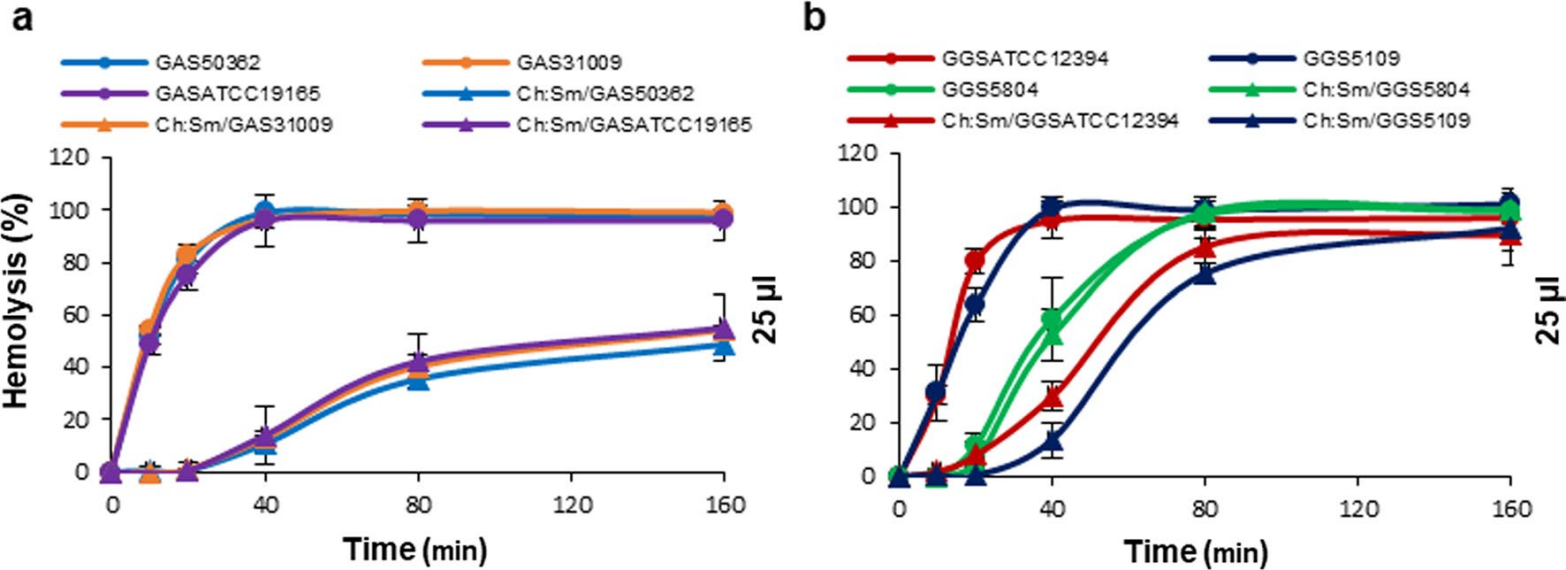
Differences in the kinetics of SLO and SLS revealed after selective inhibition of SLO. After selective inhibition of SLO by Ch:Sm-liposomes (▲), the remaining hemolytic activities of SLS develop much slower than the total hemolytic activities of the untreated GAS supernatants (●) (**a**). A similar (but smaller) lag in the development of hemolytic activities is observed for GGS 5109 and GGS ATCC 12394 supernatants (**b**). No differences in the kinetics of hemolysis between Ch:Sm-treated (▲) and untreated (●) supernatants are observed for the GGS 5804 strain (**b**). Error bars = Mean ± SD. N = 3

The two toxins also differed in their kinetics (Fig. 7). Streptococcal strains expressing significant SLO activity at their saturating amounts required 40 min to carry out complete hemolysis, whereas, after inhibition of SLO by cholesterol-containing liposomes, the remaining SLS activity developed much slower, requiring 80–160 min to reach its full extent. In GAS strains, the SLS activity effected merely 50% hemolysis even after 160 min of incubation (Fig. 7a), whereas GGS strains were capable of full hemolysis within this time interval (Fig. 7b). No difference in the kinetics of hemolysis in the presence or absence of cholesterol-containing liposomes was observed for the GGS 5804 strain, which relies almost exclusively on SLS for its hemolytic activity (Fig. 7b).

**Fig. 8:**
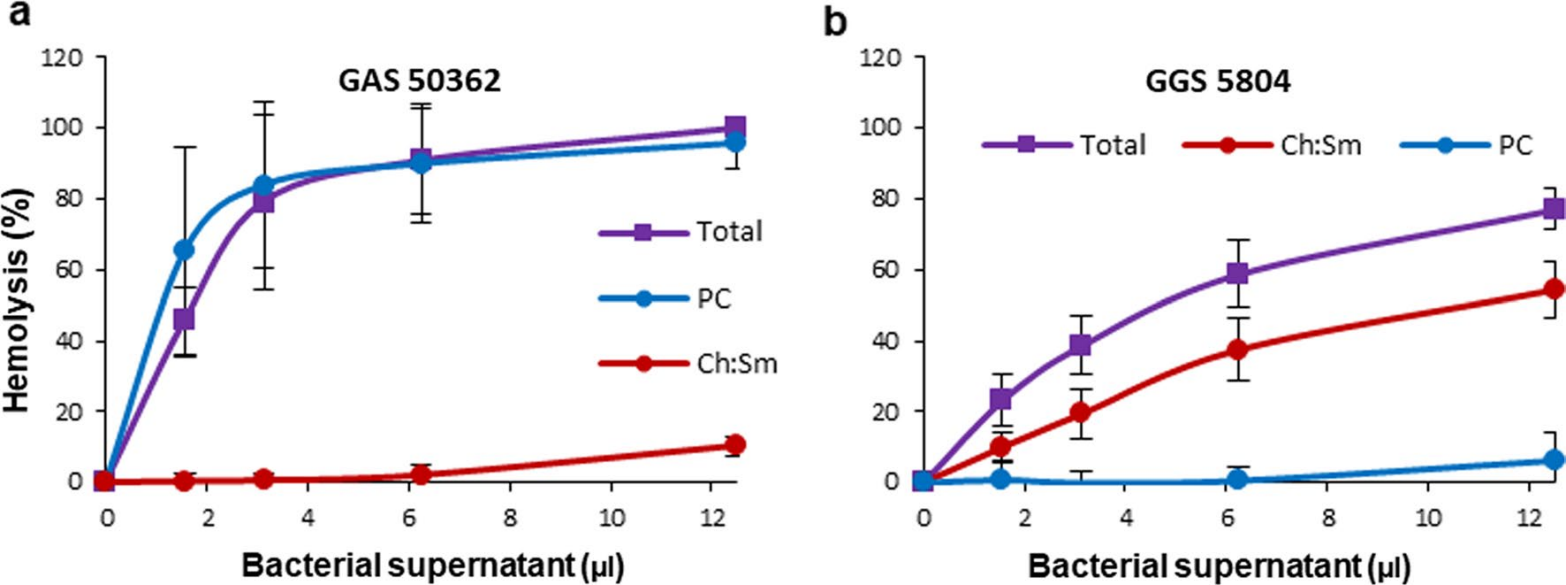
Liposome pull-down assay reveals selective removal of SLO by Ch:Sm-liposomes and SLS by 18:1/18:1 PC-liposomes from GAS 50362 (**a**) or GGS 5804 (**b**) supernatants. Total: hemolytic activity of the supernatants centrifuged in the absences of liposomes. PC or Ch:Sm: remaining hemolytic activities of the supernatants pre-treated with 18:1/18:1 PC-liposomes or Ch:Sm-liposomes before centrifugation. Error bars = Mean ± SD. N = 3

Ch:Sm-liposomes neutralize SLO by direct binding (Fig. 2c) [7, 8, 12, 13, 15]. In order to evaluate whether choline-containing liposomes neutralize SLS in a similar manner, we performed a liposomal pull-down assay using 18:1/18:1 PC-liposomes and SLS-containing GGS 5804 supernatant, whereas Ch:Sm liposomes and SLO-containing GAS 50362 supernatant served as controls (Fig. 8). As expected for the mostly SLO-containing GAS 50362 supernatant, pre-incubation with Ch:Sm–liposomes, followed by the removal of the liposomes (and liposome-bound SLO) by ultracentrifugation, almost completely abolished its hemolytic activity, whereas PC-containing liposomes provided virtually no protection (Fig. 8a). In contrast, for the mostly SLS-containing GGS 5804 supernatant, SLO-binding Ch:Sm liposomes provided little protection, whereas PC-liposomes efficiently inhibited the hemolytic activity by binding and physically removing SLS from the supernatant (Fig. 8b).

**Fig. 9:**
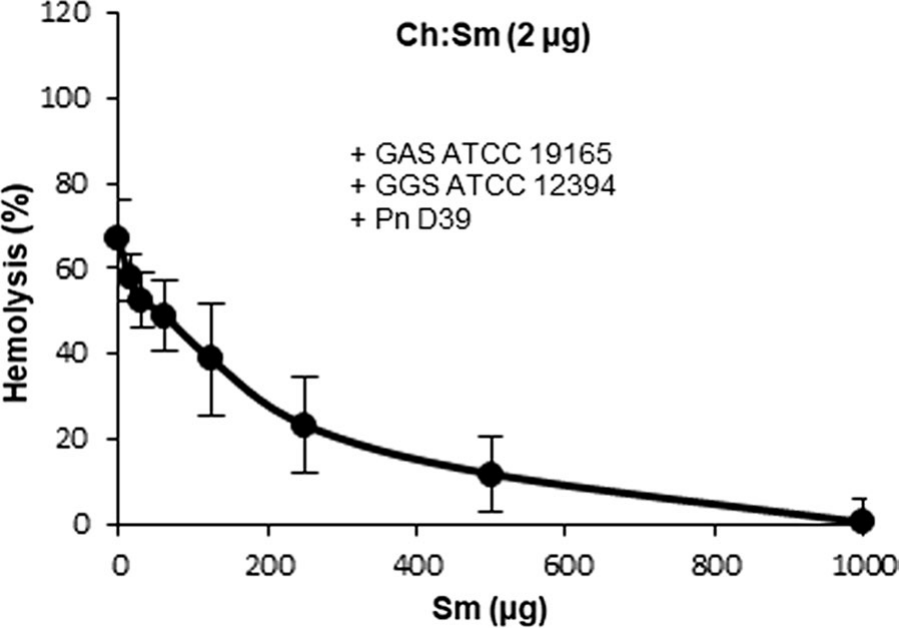
The mixture of Ch:Sm-liposomes (2 µg at any experimental condition) and Sm-liposomes (variable amount) is fully protective against the combined hemolytic action of *S. pneumonia* D39 (50 µl), + *S. pyogenes* ATCC 19165 (6.25 µl) + *S. dysgalactiae subspecies equisimilis* ATCC 12394 (6.25 µl), Error bars = Mean ± SD. N = 3

Finally, we addressed the question of whether a single liposomal formulation can simultaneously neutralize the combined hemolytic activities of *S. pneumoniae*, *S. pyogenes* and *S. dysgalactiae subspecies equisimilis*. Generally, PC-containing liposomes (two acyl chains) showed a tendency of being more efficient against SLS than Sm-containing liposomes (acyl chain and sphingosine); however, the differences within a factor of two were deemed not sufficient for practical application and further investigations. Furthermore, since PC-containing liposomes, have a significantly shorter half-life in blood than Sm-containing liposomes, the latter seem to be much better candidates for the further development as therapeutic agents [28–30]. Figure 9 shows that a combination of Ch:Sm (2 µg) and Sm-liposomes (1000 µg) was fully protective against the combination of *S. pneumoniae*, *S. pyogenes* and *S. dysgalactiae subspecies equisimilis* supernatants, each of them used at their saturating amounts that, when used separately, were capable of complete hemolysis.

## Discussion

Streptococcal pathogens are associated with life-threatening pneumonia and sepsis and cause a large variety of milder conditions [1].

Streptococcal pathogenesis is largely associated with secreted cytolysins that perforate the plasma membrane of host cells [31, 32]. Irrespective of their initial binding targets, which may be proteins, carbohydrates or lipids, cytolysins must finally interact directly with plasmalemmal lipids in order to compromise the plasmalemmal permeability barrier [12, 13, 31, 32]. Choline-containing sphingomyelin and phosphatidylcholine as well as cholesterol are major lipids of the outer leaflet of the mammalian lipid bilayer [27, 33, 34]. In contrast, bacterial membranes are composed mostly of phosphatidylethanolamine, phosphatidylglycerol and cardiolipin [34]. Therefore, eukaryote-specific lipids represent obvious targets for bacterial cytolysins in order to prevent self-harm to bacterial cells. Here, we show that liposomal nanotraps composed of eukaryote-specific lipids are capable of neutralizing the whole palette of cytolysins secreted by streptococcal pathogens.

We confirm that low doses of liposomes saturated with cholesterol efficiently neutralize CDCs [12, 13, 15]. We further confirm that PLY is the major cytolysin of α-hemolytic *S. pneumoniae* [12, 13]. Consequently, hemolytic activity of all *S. pneumoniae* strains was fully neutralizable by sub-microgram doses (low micromolar concentrations) of liposomes saturated with cholesterol.

In contrast, β-hemolytic *S. pyogenes* and *S. dysgalactiae subspecies equisimilis* secrete two hemolysins (SLO and SLS), though their relative contribution towards total hemolytic activity is highly species- and strain-dependent. Generally, SLO activity is prevalent in *S. pyogenes*, whereas the contribution of SLS is more prominent in *S. dysgalactiae subspecies equisimilis*. Due to the presence of SLS, and since only CDCs are neutralizable by cholesterol, low doses of Ch-containing liposomes provided only partial protection against either GAS or GGS. Thus, for the neutralization of these two pathogens the liposomal formulations were adapted to accommodate in addition for the activity of SLS.

We show that SLS binds to choline-containing PC and Sm. The length and the saturation of acyl chains did not influence the binding of SLS to choline-containing phospholipids.

As expected, when used alone, PC- or Sm-containing liposomes that inhibit SLS but not SLO provided only limited protection against GAS and GGS. Likewise, when used alone, low doses of Ch-containing liposomes that inhibit SLO but not SLS were only partially protective. However, the combination of low doses of Ch-containing liposomes (SLO sequestration) with liposomes composed solely of either PC or Sm (SLS sequestration) resulted in the complete neutralization of both GAS and GGS hemolytic activities. When combined in single liposome, cholesterol and PC/Sm were capable of the complete neutralization of hemolytic activities of GAS and GGS at doses similar to those of the two-liposome mixture.

Binding of CDCs to cholesterol is well documented [12, 13]. Unexpectedly, we found that CDCs also bind to fully saturated lipids, albeit with much lower efficacy. This interaction was highly specific, since the introduction of an unsaturated acyl chain at either position sn1 or sn2 of the phosphatidylcholine entirely abolished binding of CDC. From a therapeutic point of view, this interaction is of low importance due to the high doses of liposomes required for the neutralization of CDCs. However, this finding points towards an active role of lung surfactant, which is unusually rich in lipids composed of fully saturated acyl chains [35], as a protective mechanism against streptococcal infections.

The dependence of the hemolytic activities of two streptococcal hemolysins on their concentrations was non-linear. The concentration curve of SLO was sigmoidal, which is in line with its mode-of-action through the highly cooperative formation of oligomeric transmembrane pores [12, 13]. The mode of SLS action is not yet fully understood [25, 36]. The logarithmic concentration curve obtained for SLS suggests a non-cooperative mode of action, which implies that membrane destabilization by this toxin does not rely on the co-operative assembly of SLS oligomers. The two toxins also differ in their kinetics. It should be noted that our findings relating to comparative dynamics and kinetics of SLO *versus* SLS, and their individual contribution towards the total hemolytic activities of any particular streptococcal strain might only reflect the particular experimental conditions used in this study (optimal in vitro growth conditions for both GAS and GGS strains). In vivo, the concentrations and the relative contribution of the two toxins will most likely differ depending on the site of infection and other factors defined by host–pathogen interactions. However, our data emphasize that tailored liposomal nanotraps can efficiently neutralize either toxin.

Finally, we show that the mixture of Ch-containing liposomes and liposomes composed exclusively of choline-containing phospholipids was fully protective against the combined action of *S. pneumoniae*, *S. pyogenes* and *S. dysgalactiae subspecies equisimilis*. This is of high clinical relevance since these pathogens are the most frequent causative agents of sepsis [1, 14].

The burden of antibiotic-resistant bacteria is increasing in the population, which is becoming more susceptible to those organisms, and possesses fewer effective treatment options [2, 3, 37]. Antibiotics target processes that are essential for bacterial growth and survival. Consequently, they stimulate bacterial evolution and elicit the development of multidrug resistance. In recent years, resistance to new antibiotics has been reported already within 2 years or less after the introduction of a novel drug [2, 3, 37].

In contrast to classic antibiotic approaches, anti-toxin therapy aims to disarm bacteria by targeting their offensive virulence factors, exotoxins [2, 15]. Due to their near universal presence in bacteria, exotoxins are attractive targets for antimicrobial prophylaxis and therapeutics. Until recently, the antitoxin strategies were almost exclusively restricted to antibody-neutralization. Prominent examples comprise monoclonal antibodies, which target α-hemolysin of *S. aureus* (suvratoxumab) and toxin B of *Clostridium difficile* (bezlotoxumab) [4, 5]. However, these antibodies have clear limitations. By their very nature, monoclonal antibodies are highly specific: be it for a single toxin, or a single epitope of a single toxin secreted by a single bacterial serotype. Therefore, they neither address the existing, vast heterogeneity of bacterial toxins nor the fact that individual toxins are produced to varying extent during different stages of bacterial infection.

We and others have addressed the potential of anti-virulence therapy using lipid-based nanoparticles for the sequestration of membrane-damaging bacterial exotoxins [15, 19]. In contrast to highly specific, and therefore very narrow antibody-based anti-virulence strategies, the liposomal nanotraps are designed to neutralize a large variety of exotoxins belonging to different toxin families that are produced by a broad spectrum of Gram-positive and Gram-negative bacteria. Rather than individually targeting a multitude of particular exotoxins, the approach focuses on a few mechanisms by which toxins attack host cells. Since anti-toxin therapy merely targets bacterial virulence factors, the likelihood of eliciting drug resistance is low. The small and empty liposomal nanotraps are non-immunogenic and biologically neutral [15]. Their individual lipid components, which are ubiquitous, naturally occurring dietary lipids, have already been used in other pharmaceutical formulations and are proven to be non-toxic in humans. Another important benefit of the liposomal anti-virulence approach is that it does not affect the beneficial bacteria of the human microbiome. The microbiome plays an increasingly recognizable role in the human well-being, in particular in the shaping of the immune system [38, 39].

## Conclusions

Our investigation paves the way for a broad-spectrum anti-toxin therapy that fills an important medical gap as it can be applied without diagnostic delay, either as a stand-alone or as adjunct therapy to antibiotic treatment. Applied in combination with antibiotics, it might prevent the adverse effects of massive, antibiotic-induced release of bacterial toxins, and thereby markedly improve outcome. As a stand-alone therapy during mild or chronic infections, liposomal toxin-sequestration would abrogate the adverse effect of antibiotics on the host microbiome and prevent further development of antimicrobial resistance.

## Materials and methods

### Bacterial culture

Bacterial culture supernatants were prepared from *S. pyogenes* strains 19165 (ATCC, USA), 31009 (clinical isolate from blood), 50362 (clinical isolate from a biopsy), *S. dysgalactiae subspecies equisimilis* 12394 (ATCC), 5109 (clinical isolate, necrotizing fasciitis), 5804 (clinical isolate, septic arthritis) and *S. pneumoniae* D39 [40]. Bacteria were grown overnight on brain heart infusion (BHI) (Sigma-Aldrich, USA) agar plates, resuspended in BHI with 10% fetal bovine serum (FBS) (Seraglob, Switzerland) overnight at 37 °C. The culture was diluted 1:100 in BHI-FBS (10%) and incubated at 37 °C to an OD_540_ of 1. Bacterial cultures were centrifuged at 4000 rpm for 40 min at 4 °C. Culture supernatants were filtered through a 0.45 µm filter (Sarstedt, Germany), pH adjusted to 7, aliquoted and stored at − 80 °C until further use.

### Liposomal nanotraps

Egg sphingomyelin, soy phosphatidylcholine, egg phosphatidylcholine, 18:0/18:0 PC, 18:1/18:0 PC, 18:0/18:1 PC, 20:0/20:0 PC, 16:0/16:0 PC and Ch were purchased form Avanti Polar Lipids (USA) in a powder form. Liposomes were produced by sonication (20 min on ice; 5 × 10% cycles at maximal power (Bandelin Sonoplus, Germany) and kept at 4 °C until further use, as previously described [15]. Diameter of liposomes was measured by NanoSight NS300 (Malvern Panalytical, United Kingdom) for Ch:Sm-liposomes (108 ± 7 nm), Sm-liposomes (133 ± 6 nm), sPC-liposomes (152 ± 11 nm), 18:0/18:0 PC-liposomes (139 ± 2 nm), and 18:1/18:1 PC-liposomes (130 ± 9 nm). Liposome amounts correspond to the amount of total lipids used for their preparation.

### Hemolysis assay

Bacterial supernatants were serially diluted (step 2, PBS) in 96 well plates and mixed 1:1 with a 2% suspension of erythrocytes (Interregionale Blutspende SRK AG Bern, Switzerland) in PBS (final reaction volume = 200 µl). In the protection experiments, serial dilutions (step 2, PBS) of liposomal nanotraps or Trypan Blue (Thermo Fisher, USA) were added to the erythrocytes. The hemolytic reaction was initiated by adding a fixed volume of bacterial supernatant or recombinant SLO/PLY, produced as described previously (final reaction volume = 200 µl) [16, 41]. The mixture was incubated for 2 h at 37 °C and centrifuged 5 min at 4000 rpm. The supernatant was discarded and the pellet subsequently lysed using dH2O. Absorbance at 450 nm was recorded using a microplate reader (ELx808, BioTek, USA) to quantify the remaining hemoglobin. Controls consisted of a 0% hemolysis condition (PBS only) and a 100% hemolysis condition (dH2O only). Percent lysis was determined by normalizing absorbance values to the dH2O positive control (100% lysis) adjusted to the 0% lysis PBS negative control.

### Liposomal pull-down assay

For the toxin-binding assay, bacterial supernatants were pre-cleaned by ultracentrifugation at 100,000*g* for 2 h, in order to remove insoluble material.

For the detection of SLO by Western Blotting, pre-cleaned supernatants (625 µl) and Ch:Sm-liposomes (200 µg) were diluted in PBS (5 ml) and incubated for 15 min at 37 °C. Liposomes were pelleted by ultracentrifugation at 100,000*g* for 2 h. The pellets were re-suspended in 100 µl of PBS and analyzed by Western blot analysis.

For the evaluation of SLS-liposome binding, pre-cleaned supernatants (1 ml) of SLS-producing GGS 5804 strain and SLO-producing GAS 50362 strain were pre-incubated (15 min at 37 °C) with 18:1/18:1 PC-liposomes (16 mg) or Ch:Sm-liposomes (100 µg). Liposomes were pelleted by ultracentrifugation at 100,000*g* for 2 h and the hemolytic activities of the resulting, liposome-free supernatants were analyzed by hemolysis assay.

### Western blotting

Equal amounts of bacterial supernatants (7.5 µl), each harvested after reaching precisely OD_540_ of 1, were loaded on SDS-PAGE gel. Equal loading (total protein) was verified by Coomassie Blue staining.

Liposome-bound SLO or SLO from complete supernatant was detected by Western blot. Recombinant SLO and BHI 10% FBS medium were used as positive and negative control respectively. Immunoblotting was performed with a polyvinylidene difluoride membrane (Sigma-Aldrich, USA). A rabbit polyclonal antibody against SLO (Bio Academia, Japan) was used at 1:2000 dilution. A goat anti-rabbit IgG horseradish peroxidase-linked (BD Bioscience, USA), diluted 1:1000, was used as secondary antibody. The membrane was developed with WesternBright ECL (Advansta, USA), read by Fusion FX (Vilber Lourmat, France), the band intensities were analyzed by FIJI [42]. The statistical analysis was performed with GraphPad Prism (USA) using a one-way ANOVA followed by post hoc Tukey HSD test.

## Data Availability

All data generated or analyzed during this study are included in this published article.
